# Diamine Oxidase Activity Deficit and Idiopathic Rhinitis: A New Subgroup of Non-Allergic Rhinitis?

**DOI:** 10.3390/life13010240

**Published:** 2023-01-14

**Authors:** Miguel Mayo-Yáñez, Andrea Díaz-Díaz, Christian Calvo-Henríquez, Jerome R. Lechien, Luigi A. Vaira, Angélica Figueroa

**Affiliations:** 1 Otorhinolaryngology—Head and Neck Surgery Department, Complexo Hospitalario Universitario A Coruña (CHUAC), 15006 A Coruña, Spain; 2 Clinical Research in Medicine, International Center for Doctorate and Advanced Studies (CIEDUS), Universidade de Santiago de Compostela (USC), 15782 Santiago de Compostela, Spain; 3 Epithelial Plasticity and Metastasis Group, Instituto de Investigación Biomédica de A Coruña (INIBIC), Complexo Hospitalario Universitario de A Coruña (CHUAC), Universidade da Coruña (UDC), 15006 A Coruña, Spain; 4 Otorhinolaryngology—Head and Neck Surgery Department, Complexo Hospitalario Universitario Santiago de Compostela (CHUS), 15706 Santiago de Compostela, Spain; 5 Division of Laryngology & Broncho-Esophagology, EpiCURA Hospital, University of Mons, 7331 Baudour, Belgium; 6 Department of Otolaryngology-Head & Neck Surgery, Foch Hospital, School of Medicine, UFR Simone Veil, Université Versailles Saint-Quentin-en-Yvelines (Paris Saclay University), 91190 Paris, France; 7 Department of Otolaryngology, Elsan Hospital Poitiers, 86000 Poitiers, France; 8 Department of Human Anatomy and Experimental Oncology, Faculty of Medicine, UMONS Research Institute for Health Sciences and Technology, University of Mons (UMons), 7000 Mons, Belgium; 9 Maxillofacial Surgery Operative Unit, Department of Medicine, Surgery and Pharmacy, University of Sassari, 07100 Sassari, Italy; 10 Department of Biomedical Science, PhD School of Biomedical Science, University of Sassari, 07100 Sassari, Italy

**Keywords:** idiopathic rhinitis, diamine oxidase, histamine, TRPV1, peak nasal inspiratory flow, rhinology, maxillofacial surgery, otorhinolaryngology

## Abstract

Idiopathic rhinitis represents more than 50% of non-allergic rhinitis, a heterogeneous group that involves the symptomatic inflammation of the nasal mucosa. The TRPV1 receptor of unmyelinated C-type neurons appears to be involved in its pathophysiology. Histamine, whose main catabolic enzyme is DAO, is one of the mediators that can activate this receptor. The failure of DAO causes an increase in the level of histamine in the body and, consequently, the activation of TRPV1. The objective was to investigate the existence of a DAO enzyme activity deficit in idiopathic rhinitis and its correlation with symptoms. A cross-sectional study was conducted in 116 idiopathic rhinitis patients, and DAO activity, nasal peak inspiratory flow, and rhinitis severity were recorded. The prevalence of a DAO activity deficit was 41.38% (95%CI 0.33–0.50; *p* = 0.05). The DAO activity in patients with mild rhinitis was 52.93 ± 8.72 HDU/mL, in those with moderate rhinitis it was 120.33 ± 71.63 HDU/mL, and in those with severe rhinitis it was 92.58 ± 27.75 HDU/mL (*p* = 0.006). The NPIF in patients with a DAO activity deficit was 107.92 ± 34.05 L/min, compared to 72.35 ± 27.16 L/min in patients with normal enzymatic activity (*p* < 0.001), demonstrating a linear correlation between activity levels and nasal obstruction (−0.45; *p* < 0.001). Therefore, patients with a DAO deficiency and idiopathic rhinitis could present a milder disease course, because the repeated and continuous activation of TRPV1 led to a partial or total decrease in their response (desensitization). This new theory represents a different perspective for the study of idiopathic rhinitis and its relationship with TRPV1, with the regulation or modulation of the desensitization of TRPV1 being an important therapeutic target for patients with idiopathic rhinitis in the future.

## 1. Introduction

Rhinitis is defined as a symptomatic inflammation of the inner lining of the nose, classically divided into allergic rhinitis (AR) and non-allergic rhinitis (NAR). NAR comprises a large heterogeneous group of patients without systemic signs of allergic inflammation (allergen-specific IgE in the blood and/or positive SPT results) or clinical signs of infection. Of these cases, up to 50% do not have a clear etiology underlying the symptoms and are defined as idiopathic rhinitis (IR) [1,2,3].

Several mechanisms have been postulated to explain IR pathophysiology. One of the most widely accepted suggestions is the nociceptive transient receptor potential vanilloid 1 (TRPV1) signaling pathway, which is found upregulated in IR patients [4,5]. Within the sensory nerve fibers present in organs, TRPV1 is implicated in the detection and integration of thermal and chemical nociceptive stimuli [6]. Moreover, TRPV1 is particularly widely distributed in the sensory fibers of the upper and lower respiratory tracts [7,8,9,10,11].

**Figure 1:**
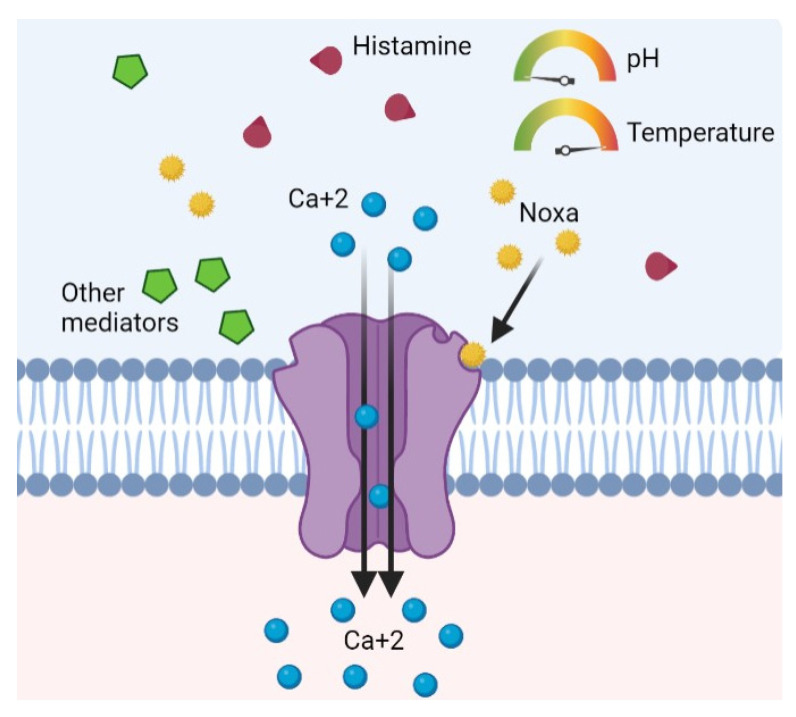
TRPV1 receptor of unmyelinated sensory C fibers is activated by several physiological stimuli, exogenous noxa, or endogenous inflammatory mediators.

This receptor can be activated, directly or indirectly, by a large number of mediators: ethanol, tissue damage, mechanical stimuli, acid pH, temperature above 43 °C, osmotic pressure alterations, cations, leukotrienes, nitric oxide, lipoxygenases, capsaicin, substance P, nerve growth factors, and prostaglandins (Figure 1) [6,7,12,13,14,15,16,17]. Curiously, another mediator capable of activating TRPV1 is histamine, which is the most studied mediator of AR [16].

Histamine intolerance (HIT), an imbalance between the supply of histamine and the body’s ability to degrade it, belongs to the group of pharmacological non-IgE-mediated food intolerances. Its prevalence in the general population is approximately 1%, but this figure is probably underestimated due to the condition’s wide spectrum of symptoms [18,19,20,21]. Ingested exogenous histamine is distributed in the blood stream and may trigger a wide range of symptoms in the susceptible population. Its main cause is a deficit in histamine degradation due to a genetic, pathological, or pharmacological malfunction of the diamine oxidase (DAO) enzyme, which can cause an increase in the plasma concentration of histamine. The DAO enzyme represents the main enzyme involved in histamine metabolism [18,19,20,21], and its activity can be influenced by numerous factors. DAO gene transcription or enzyme function may be reduced in certain gene polymorphisms [20,22,23]. Recent clinical studies have established a relationship between DAO malfunction and specific pathologies, mainly dermatological, respiratory (including AR), gastrointestinal, and neurological [20,22,23,24,25,26,27,28,29,30]. However, no studies have addressed the relationship between a DAO enzymatic activity deficit and IR and its correlation with symptoms.

The physiology of the TRPV1 receptor and its response to histamine could allow for a subgroup of patients with IR and a DAO activity deficit in which the symptomatology and clinical course vary; therefore, these patients could benefit from personalized treatment. The objective of this preliminary study was to investigate the existence of a DAO enzyme activity deficit in patients diagnosed with IR and its correlation with symptoms.

## 2. Materials and Methods

A cross-sectional study involving patients diagnosed with IR according to the European Academy of Allergy and Clinical Immunology criteria [1] was performed in an otorhinolaryngology head and neck surgery department of a tertiary university hospital.

Candidate patients were required to present at least two of the following symptoms for at least 1 h daily for 12 weeks per year: nasal obstruction, rhinorrhea (anterior or posterior), sneezing, and nasal/ocular itch. Patients with rhinosinusitis and occasional or nasal symptoms were excluded. Selection was based on a structured interview and physical examination (anterior rhinoscopy and flexible fibronasoendoscopy) revealing no evidence of important nasoseptal deformities or polyps, performed independently by at least one otorhinolaryngologist and one allergist. For each participant, a detailed history of allergies was collected, including a skin-prick test for common aeroallergens. The relevant subgroups of NAR in clinical practice (rhinitis of the elderly, drug-induced rhinitis, hormonal rhinitis including nonallergic occupational rhinitis, pregnancy-induced rhinitis, and gustatory rhinitis) were excluded based on this clinical history, with support from the necessary complementary tests as appropriate. The key feature of IR is the presence of nasal hyperresponsiveness [1]. Since there are no validated methods for the evaluation of nasal hyperresponsiveness that can distinguish its etiology [31], the diagnosis of IR was carried out per exclusionem, on the basis of the symptoms reported by the patients and the absence of a clinical history that could lead us to suspect another possible etiology.

Several diagnostic strategies involving various tests have been proposed, because direct HIT-specific diagnostic criteria are lacking. Following the recommendations of the clinical guidelines, only cases matching the analytical criteria and presenting a compatible clinical history (history of flushing, itching, diarrhea, nausea/vomiting, abdominal pain, dyspnea, rhinitis, dysphonia, dizziness, low blood pressure, or tachycardia) were classified as DAO deficiency [20,21,32,33,34]. It was necessary to analyze the symptoms with reference to their temporal onset for the differential diagnosis, because adverse food reactions are only suspected in the case of a temporal relationship (min <4 h) to food intake.

Only adults over 18 years old were included. The exclusion criteria were: pregnancy; treatment with the DAO enzyme for the past 2 weeks; treatment with any medication that may have caused a secondary decrease (antihistamines, aminophylline, systemic corticosteroids, cefuroxime, verapamil, metoclopramide clavulanic acid, etc.) or increase (heparin) in DAO activity and could not be suspended [18,19,21]; a history of histamine-rich aliment intake in the past 2 weeks (fermented foods, beverages, processed meat or seafood); and medical disorders (severe hepatic, renal, or gastrointestinal disease; zinc, copper, and vitamin B6 or C deficiency, etc.) that could alter the analytical results.

### 2.1. Data Collection

Sociodemographic variables; DAO enzyme activity; nasal peak inspiratory flow; and IR severity score (mild 0–3, moderate 4–7, and severe 8–10) based on a visual analogue scale (VAS, 0–10 cm calibrated) were recorded [34,35].

Nasal peak inspiratory flow (NPIF) is a rapid and simple technique performed using a plastic cylinder calibrated between 30 and 370 L/min with a facial mask attached. In this study, we used the In-Check portable inspiratory flow meter (Clement Clarke International Ltd., Harlow, UK). From an expiratory maneuver to residual volume, a forced inspiration was made while the lips were sealed. The best of three measurements that varied by no more than a 10% was chosen. Normal reference values for Caucasian adults are 143 ± 48.6 L/min for males and 121.9 ± 36 L/min for females [35,36].

DAO enzyme activity was determined by ELISA in accordance with the manufacturer’s instructions (D-HIT, Sciotec, Donau, Austria) and previous literature reports, with a venipuncture after an 8 h fasting period in an EDTA tube [37]. The threshold for serum DAO enzyme activity has been proposed to be 80 HDU/mL, with a value below this figure indicative of a DAO activity deficiency; in such a case, the presence of HIT should be considered. One HDU corresponds to the DAO activity needed to degrade 1 pmol/mL (0.11 ng/mL) of histamine. The usefulness of DAO measurement in HIT as a marker of disease and disease severity and as a predictor of treatment response has been validated in several studies [38,39].

In order to exclude patients with allergies, a skin-prick test with a calibrated lancet (1 mm) held vertically while introducing a drop of diluted purified allergen was used. According to the protocol of the Hospital’s Allergology Service, and based on the allergens typical of the area in which the study was carried out, the extracts included were Dermatophagoides pteronyssinus, Lepidoglyphus destructor, Tyrophagus putrescentiae, Gliciphagus, Blomia tropicalis, Chortogliphus, Alternaria alternata, Phleum pratense, Cynodon dactilon, Plantago lanceolata, Parietaria judaica, Betula verrucosa, Populus nigra, Cupressus arizonica, Platanus acerifolia, latex, profilina, and dog and cat epithelium. A drop of histamine (10 mg/mL) and saline solution were used as a positive and negative control, respectively. The maximum or mean diameters of the wheals for various allergens were read at 15 min. A wheal ≥2 mm in diameter was considered positive, indicating sensitization to the allergen.

### 2.2. Statistical Analysis

Statistical analysis was performed with R program 3.6.1 (The R Foundation for Statistical Computing^®^, Vienna, Austria). Normality was evaluated by the Kolmogorov–Smirnov test and variances using the Levene test. All tests were two-tailed with a 95% confidence interval (CI). Quantitative variables were expressed as mean ± standard deviation (SD) and/or median, as appropriate. For the qualitative variables, frequency and percentage were used. The comparison of means was performed using Student’s *t* test, while qualitative or categorical differences between groups were evaluated by the χ^2^ test or Fisher’s exact test, as appropriate. In cases where non-normality was significant, non-parametric methods were applied. Correlation between variables was assessed using Pearson’s test or Spearman’s test, as appropriate.

## 3. Results

A total of 116 Caucasian patients with IR were recruited: 20 men (17.24%) and 96 (82.76%) women. The mean age was 45.36 ± 11.41 years (men: 35.61 ± 15.96 years; women: 47.39 ± 9.08 years). The prevalence of DAO activity deficits in patients with IR was 41.38% (95%CI, 0.33–0.50). The mean DAO enzyme activity of the 116 patients was 108.98 ± 63.9 HDU/mL: 85.60 ± 43.15 HDU/mL in men and 113.85 ± 66.57 HDU/mL in women (*p* = 0.161). The mean DAO activity in the normal-function group (i.e., patients with DAO activity ≥80 HDU/mL) was 143.05 ± 63.96 HDU/mL, compared to 60.72 ± 10.16 HDU/mL in the group with an activity deficit (*p* < 0.000).

According to the severity of the symptoms measured using the VAS, 8 (6.90%) patients had “mild” rhinitis, 80 (68.97%) “moderate” rhinitis, and 28 (24.14%) “severe” rhinitis. The global mean was 6.35 ± 1.78 points, being 5.6 ± 2.3 for men and 6.51 ± 1.63 for women *(p =* 0.393). After stratification according to severity groups, the mean score was 2 in the mild IR group, 6.05 ± 0.89 in the moderate IR group, and 8.50 ± 0.73 in the severe IR group (*p* < 0.001). The DAO activity in patients with mild rhinitis was 52.93 ± 8.72 HDU/mL, in moderate rhinitis it was 120.33 ± 71.63 HDU/mL, and in severe IR it was 92.58 ± 27.75 HDU/mL (*p* = 0.006).

**Figure 2:**
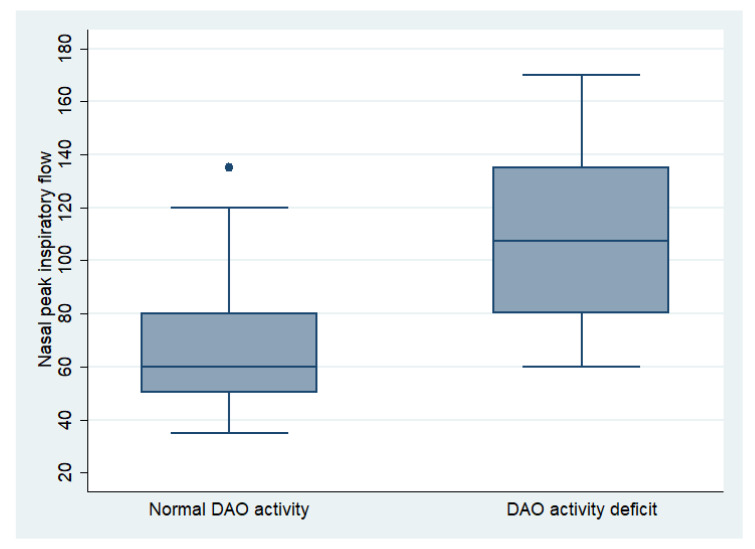
Comparation of nasal peak inspiratory flow between patients with normal DAO activity and DAO activity deficit.

The mean NPIF of all patients was 87.06 ± 34.82 L/min, being 89 ± 26.23 L/min for men and 86.67 ± 36.46 L/min for women (*p* = 0.514). In the mild group, the NPIF was 85 ± 10.69 L/min, in the moderate group it was 88.25 ± 37.29 L/min, and in the severe group it was 84.28 ± 32.48 L/min (*p* = 0.904). The NPIF in patients with a DAO activity deficit was 107.92 ± 34.05 L/min, compared to 72.35 ± 27.16 L/min in patients with normal enzymatic activity (*p* < 0.001) (Figure 2), demonstrating a linear correlation between activity levels and nasal obstruction (r = −0.45; *p* < 0.001).

## 4. Discussion

HIT, also known as sensitivity to dietary histamine or enteral histaminosis, can be defined as a dysfunction caused by a reduction in DAO activity that affects the degradation of histamine in the intestine. Under physiological conditions, DAO creates an intestinal enzymatic barrier that protects the body from the absorption of histamine ingested with food [19,20]. HIT provokes a wide range of symptoms (extraintestinal and gastrointestinal) due to the fact that histamine receptors are widespread in all tissues of the body. This amount of histamine is usually well-tolerated in healthy individuals [18,19].

IR represents a diagnostic and therapeutic challenge [1]. The mechanisms involved in its pathophysiology are still unknown, but the TRPV1 receptors have become more relevant, since the activation of this ion channel causes the release of the calcitonin G-related and substance P neuropeptides from nerve terminals, causing a local inflammatory reaction. Several publications have established a connection between TRPV1 activation in the airways and asthma or NAR [6,7]. The activation of the unmyelinated C-fibers where these receptors are present provokes bronchoconstriction; mucus secretion; bradycardia; itching; pain; the irritation of the airways; vasodilation; edema; and, due to the chemotactism, the recruitment, differentiation, and activation of many immune cells (eosinophils, lymphocytes, macrophages, and mast cells). This inflammatory reaction is called “neurogenic inflammation” [40].

The activity and efficiency of TRPV1 under physiological conditions are low. An inflammatory process activates peripheral sensory nociceptors that are capable of locally releasing numerous pro-algesic chemical mediators, such as histamines, serotonins, bradykinins, prostaglandins, cytokines, and growth factors. All of these contribute to enhancing the local inflammation process, and consequently, the function of TRPV1 is synergistically enhanced by the simultaneous action of these released pro-inflammatory substances. These substances cause an increase in the sensitivity and response of peripheral nociceptors, which leads to a decrease in the activation threshold of these terminals and an increase in the magnitude of their response. This phenomenon is known as peripheral sensitization [41,42].

**Figure 3:**
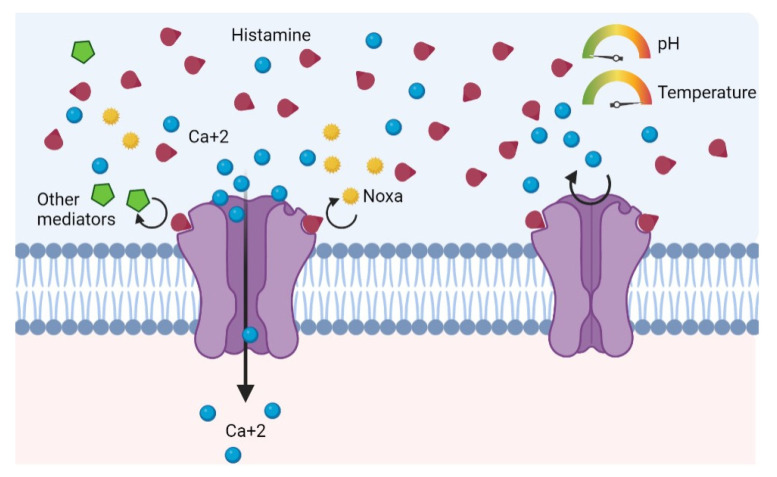
Continuous activation and consequent refractory (desensitization) due to histamine action as a competitive agonist.

One particularity of TRPV1 is that the repeated and continuous activation of nociceptors leads to a partial or total decrease in their response. The TRPV1 becomes refractory, being unable to respond to new stimuli (Figure 3) [10,43]. This state is known as desensitization [44,45]. Likewise, prolonged exposure to agonists, such as histamine in this case, promotes the endocytosis of the receptor and subsequent lysosomal degradation, perpetuating the state of desensitization and decreased response to stimuli [46]. One possibility is that the increase in circulating histamine due to HIT causes an increase in vasodilation and chemotactism locally in the nasal mucosa, facilitating the release of histamine from mast cells. This in turn would activate or sensitize primary afferent C-fibers via TRPV1 [13,47].

Histamine could act as a competitive agonist against other molecules, displacing a possible stronger response to the activation of TRPV1. In fact, previous studies suggest that the increase in calcium concentrations stimulated by histamine is slower, with a lower amplitude and shorter duration compared to capsaicin activation, even in the continuing presence of the agonist [48]. Furthermore, a prolonged state of desensitization could induce the endocytosis of the TRPV1 receptors, causing a decrease in the response to stimuli [10]. The repeated stimulation of TRPV1 by a non-physiological increase in histamine due to a deficit in its degradation could cause this phenomenon. Future studies are needed to clearly elucidate the potential implications of this hypothesis. In fact, contrary to what happens in acute desensitization, the pathways involved in the medium-to-long-term desensitization of the receptor have been barely studied [10].

In this study, DAO activity was investigated in patients diagnosed with IR. Other authors have studied serum or intracellular DAO concentrations [27]. Nevertheless, the present study was the first to use DAO activity and not only its concentration, thus succeeding in exploring the relationships between IR symptoms and alterations in histamine metabolism related to a DAO activity deficiency. The prevalence of DAO deficiencies found in our study was consistent with the results of other studies addressing different pathological conditions such as chronic urticaria, atopic eczema, chronic abdominal pain, and lactose malabsorption, which reported values ranging between 8% and 57% [20,22,23,24,25]. Another important aspect for future studies is the concurrent investigation of rhinitis and laryngopharyngeal reflux [49]. Indeed, some recent data suggested a potential relationship between HIT and laryngopharyngeal reflux [50], the latter possibly being involved in rhinitis.

Several authors have detected a correlation between DAO concentrations and female sex hormone changes [18], but this was not observed in our case series for DAO activity. Approximately 40% of the IR patients with a clinical history and analytical results compatible with a DAO activity deficit presented better NPIF results compared to the normal-DAO-activity group. This finding suggests the existence of a subgroup of patients diagnosed with IR whose clinical presentation differs from the rest. Past studies have assessed DAO activity, and three non-synonymous single-nucleotide polymorphisms (SNPs) have been mapped to the DAO gene (chromosome 7q34–36). Functional impairment was related to serum DAO activity only for the SNP with the refSNP ID rs1049793 (NCBI SNP database), which codes for an altered protein with the amino acid substitution His645Asp [23]. Similar studies analyzing the prevalence of DAO deficiency in AR have demonstrated the existence of this mutation in 38% of AR patients, a figure very similar to the prevalence of enzymatic impairment found in our study [3,22]. Contrary to the findings in AR patients, in whom serum DAO was positively correlated with severity [24,27,28,30], our work showed a moderate inverse correlation. This was in line with the hypothesis previously stated. This difference in AR and IR symptomatology according to DAO activity seems to be linked to the mechanism of histamine activation in each case. While the inflammatory pathway in IR would be via TRPV1, in the case of AR, it is an IgE-mediated response with Th2 activation [5].

An important limitation of this study is the impossibility of extrapolating the prevalence of DAO activity deficiencies in the general population, as the patients were recruited only from the outpatient department, and there was no control group. For this reason, it is not possible to conclude whether the IR subgroups established on the basis of DAO activity represent the variability of the general population or non-IR patients. Patients’ motivation to seek care can be influenced by symptom severity, introducing an inclusion bias. In other words, patients with very mild or very severe symptoms may not recognize them as pathological [3,51]. This may have influenced the severity distribution of IR found in the sample. Other limitations include the lack of other parameters (serum histamine, IgE) and the absence of a prospective evaluation with repeated measures of DAO activity over time [52,53]. Finally, only 17% of the patients included in the study were men, and it is possible that this introduced selection bias, affecting the results. The strengths of this study include the physiological basis previously demonstrated in the literature, on which the hypothesis was based; the evaluation of the NPIF; and the first ever investigation of the relationship between histamine intolerance and DAO activity in IR patients [31,33,36,54,55].

Although our results were not sufficient to definitively clarify the link between IR severity and DAO activity, this study opens up a new range of possibilities. No study has addressed the role of DAO SNPs in patients with IR. It can be hypothesized that, as in the case of AR, the presence of these polymorphisms may modify the pathogenesis of IR due to the involvement of DAO in the metabolism of circulating histamine.

## 5. Conclusions

The hypothesis of this manuscript was that the repeated stimulation of TRPV1 could be caused by supra-physiological histamine levels generated due to a deficit in their degradation, producing a less severe symptomatology in a subgroup of patients with IR. The results of the pilot study supported this claim: patients with mild rhinitis had lower DAO activity than patients with moderate and severe rhinitis, and the decrease in DAO activity was inversely proportional to nasal obstruction assessed according to NPIF. This represents a new perspective for the study of IR and its relationship with TRPV1, with the regulation or modulation of the desensitization of TRPV1 being an important therapeutic target for patients with IR in the future.

## Data Availability

The data presented in this study are available on request from the corresponding author. The data are not publicly available due to confidentiality.

## References

[1] Hellings P.W., Klimek L., Cingi C., Agache I., Akdis C., Bachert C., Bousquet J., Demoly P., Gevaert P., Hox V. (2017). Non-allergic rhinitis: Position paper of the European Academy of Allergy and Clinical Immunology. Allergy.

[2] Avdeva K.S., Fokkens W.J., Segboer C., Reitsma S. (2022). The prevalence of non-allergic rhinitis phenotypes in the general population: A cross-sectional study. Allergy.

[3] Mayo-Yáñez M., Villares-Soriano J., Calvo-Henríquez C., Vázquez-Barro J.C., Herranz González-Botas J., Martín-Martín C. (2019). Allergic rhinitis versus Idiopathic rhinitis: Are there differences in the symptomatology and nasal obstruction?. Rev. Française D’Allergologie.

[4] Van Gerven L., Alpizar Y.A., Steelant B., Callebaut I., Kortekaas Krohn I., Wouters M., Vermeulen F., Boeckxstaens G., Talavera K., Hellings P.W. (2017). Enhanced chemosensory sensitivity in patients with idiopathic rhinitis and its reversal by nasal capsaicin treatment. J. Allergy Clin. Immunol..

[5] Van Gerven L., Boeckxstaens G., Hellings P. (2012). Up-date on neuro-immune mechanisms involved in allergic and non-allergic rhinitis. Rhinology.

[6] Du Q., Liao Q., Chen C., Yang X., Xie R., Xu J. (2019). The Role of Transient Receptor Potential Vanilloid 1 in Common Diseases of the Digestive Tract and the Cardiovascular and Respiratory System. Front. Physiol..

[7] Pattanaik D., Lieberman P. (2010). Vasomotor rhinitis. Curr. Allergy Asthma Rep..

[8] Yang X.R., Lin M.J., McIntosh L.S., Sham J.S.K. (2006). Functional expression of transient receptor potential melastatin- and vanilloid-related channels in pulmonary arterial and aortic smooth muscle. Am. J. Physiol. Lung Cell. Mol. Physiol..

[9] Seki N., Shirasaki H., Kikuchi M., Sakamoto T., Watanabe N., Himi T. (2006). Expression and localization of TRPV1 in human nasal mucosa. Rhinology.

[10] Ständer S., Moormann C., Schumacher M., Buddenkotte J., Artuc M., Shpacovitch V., Brzoska T., Lippert U., Henz B.M., Luger T.A. (2004). Expression of vanilloid receptor subtype 1 in cutaneous sensory nerve fibers, mast cells, and epithelial cells of appendage structures. Exp. Dermatol..

[11] Lundberg J.M., Saria A. (1987). Polypeptide-containing neurons in airway smooth muscle. Annu. Rev. Physiol..

[12] Holland C., van Drunen C., Denyer J., Smart K., Segboer C., Terreehorst I., Newlands A., Beerahee M., Fokkens W., Tsitoura D.C. (2014). Inhibition of capsaicin-driven nasal hyper-reactivity by SB-705498, a TRPV1 antagonist. Br. J. Clin. Pharmacol..

[13] Tominaga M., Tominaga T. (2005). Structure and function of TRPV1. Pflugers Arch..

[14] Ahern G.P., Wang X., Miyares R.L. (2006). Polyamines are potent ligands for the capsaicin receptor TRPV1. J. Biol. Chem..

[15] Ahern G.P., Brooks I.M., Miyares R.L., Wang X. (2005). Extracellular cations sensitize and gate capsaicin receptor TRPV1 modulating pain signaling. J. Neurosci..

[16] Akdis C.A., Blaser K. (2003). Histamine in the immune regulation of allergic inflammation. J. Allergy Clin. Immunol..

[17] Pingle S.C., Matta J.A., Ahern G.P. (2007). Capsaicin Receptor: TRPV1 a Promiscuous TRP Channel. Handbook of Experimental Pharmacology.

[18] Kovacova-Hanuskova E., Buday T., Gavliakova S., Plevkova J. (2015). Histamine, histamine intoxication and intolerance. Allergol. Et Immunopathol..

[19] Maintz L., Novak N. (2007). Histamine and histamine intolerance. Am. J. Clin. Nutr..

[20] Comas-Basté O., Sánchez-Pérez S., Veciana-Nogués M.T., Latorre-Moratalla M., Del Carmen Vidal-Carou M. (2020). Histamine Intolerance: The Current State of the Art. Biomolecules.

[21] Lefèvre S., Astier C., Kanny G. (2017). Intolérance à l’histamine ou fausses allergies alimentaires de mécanisme histaminique. Rev. Française D’Allergologie.

[22] Meza-Velázquez R., López-Márquez F., Espinosa-Padilla S., Rivera-Guillen M., Gutíerrez-Díaz N., Pérez-Armendáriz L., Rosales-González M. (2016). Association between two polymorphisms of histamine-metabolising enzymes and the severity of allergic rhinitis in a group of Mexican children. Allergol. Immunopathol..

[23] Garcia-Martin E., Garcia-Menaya J., Sanchez B., Martinez C., Rosendo R., Agundez J.A. (2007). Polymorphisms of histamine-metabolizing enzymes and clinical manifestations of asthma and allergic rhinitis. Clin. Exp. Allergy.

[24] Enko D., Kriegshäuser G., Halwachs-Baumann G., Mangge H., Schnedl W.J. (2017). Serum diamine oxidase activity is associated with lactose malabsorption phenotypic variation. Clin. Biochem..

[25] Rosell-Camps A., Zibetti S., Pérez-Esteban G., Vila-Vidal M., Ferrés-Ramis L., García-Teresa-García E. (2013). Histamine intolerance as a cause of chronic digestive complaints in pediatric patients. Rev. Esp. Enferm. Dig..

[26] Mayo-Yáñez M., Díaz-Díaz A., Vázquez-Barro J.C., Herranz González-Botas J., Figueroa A., Martín-Martín C.S. (2021). Relationship between allergic rhinitis and diamine oxidase activity: A preliminary report. Allergol. Select.

[27] Maintz L., Benfadal S., Allam J.P., Hagemann T., Fimmers R., Novak N. (2006). Evidence for a reduced histamine degradation capacity in a subgroup of patients with atopic eczema. J. Allergy Clin. Immunol..

[28] Izquierdo J., Mon D., Lorente M., Singla L.S. (2013). A randomized doubled blinded trial of treatment with diamino-oxidase (DAO) in patients with migraine and deficit of enzyme’s activity. J. Neurol. Sci..

[29] Refaat M.M., Abdel-Rehim A.S., Elmahdi A.R., Mohamed N.A., Ghonaim S.S. (2019). Diamine oxidase enzyme: A novel biomarker in respiratory allergy. Int. Forum Allergy Rhinol..

[30] Meza-Velázquez R., López-Márquez F., Espinosa-Padilla S., Rivera-Guillen M., Ávila-Hernández J., Rosales-González M. (2017). Association of diamine oxidase and histamine N-methyltransferase polymorphisms with presence of migraine in a group of Mexican mothers of children with allergies. Neurologia.

[31] Gerth van Wijk R.G., de Graaf-in ‘t Veld C., Garrelds I.M. (1999). Nasal hyperreactivity. Rhinology.

[32] Reese I., Ballmer-Weber B., Beyer K., Fuchs T., Kleine-Tebbe J., Klimek L., Lepp U., Niggemann B., Saloga J., Schäfer C. (2017). German guideline for the management of adverse reactions to ingested histamine: Guideline of the German Society for Allergology and Clinical Immunology (DGAKI), the German Society for Pediatric Allergology and Environmental Medicine (GPA), the German Association of Allergologists (AeDA), and the Swiss Society for Allergology and Immunology (SGAI). Allergo J. Int..

[33] Test no validados de intolerancia a alimentos: Documento de posicionamiento del Grupo Andaluz de Trastornos Funcionales Digestivos (GATFD) pertenecientes a la Sociedad Andaluza de Patología Digestiva (SAPD) y el Colegio Profesional de Dietistas-Nutricionistas de Andalucía | RAPD Online | SAPD. https://www.sapd.es/revista/2018/41/6/01.

[34] Mayo-Yáñez M., Díaz-Díaz A., Calvo-Henríquez C., Chiesa-Estomba C., Figueroa A., Martín-Martín C.S. (2021). Usefulness of the histamine intolerance assessment questionnaire for diagnosis. Rev. Française D’Allergologie.

[35] Valero A., Navarro A.M., Del Cuvillo A., Alobid I., Benito J.R., Colás C., de Los Santos G., Liesa F., García-Lliberós A., González-Pérez R. (2018). Position paper on nasal obstruction: Evaluation and treatment. J. Investig. Allergol. Clin. Immunol..

[36] Rimmer J., Hellings P., Lund V.J., Alobid I., Beale T., Dassi C., Douglas R., Hopkins C., Klimek L., Landis B. (2019). European position paper on diagnostic tools in rhinology. Rhinology.

[37] Music E., Korosec P., Silar M., Adamic K., Kosnik M., Rijavec M. (2013). Serum diamine oxidase activity as a diagnostic test for histamine intolerance. Wien. Klin. Wochenschr..

[38] Cucca V., Ramirez G.A., Pignatti P., Asperti C., Russo M., Della-Torre E., Breda D., Burastero S.E., Dagna L., Yacoub M.R. (2022). Basal Serum Diamine Oxidase Levels as a Biomarker of Histamine Intolerance: A Retrospective Cohort Study. Nutrients.

[39] Boehm T., Pils S., Gludovacz E., Szoelloesi H., Petroczi K., Majdic O., Quaroni A., Borth N., Valent P., Jilma B. (2017). Quantification of human diamine oxidase. Clin. Biochem..

[40] Hunter D.D., Myers A.C., Undem B.J. (2000). Nerve growth factor-induced phenotypic switch in guinea pig airway sensory neurons. Am. J. Respir. Crit. Care Med..

[41] Carr M.J., Hunter D.D., Jacoby D.B., Undem B.J. (2002). Expression of tachykinins in nonnociceptive vagal afferent neurons during respiratory viral infection in guinea pigs. Am. J. Respir. Crit. Care Med..

[42] Cholewinski A., Burgess G.M., Bevan S. (1993). The role of calcium in capsaicin-induced desensitization in rat cultured dorsal root ganglion neurons. Neuroscience.

[43] Smutzer G., Devassy R.K. (2016). Integrating TRPV1 Receptor Function with Capsaicin Psychophysics. Adv. Pharmacol. Sci..

[44] Koplas P.A., Rosenberg R.L., Oxford G.S. (1997). The role of calcium in the desensitization of capsaicin responses in rat dorsal root ganglion neurons. J. Neurosci..

[45] Sanz-Salvador L., Andrés-Borderia A., Ferrer-Montiel A., Planells-Cases R. (2012). Agonist- and Ca2+-dependent Desensitization of TRPV1 Channel Targets the Receptor to Lysosomes for Degradation. J. Biol. Chem..

[46] Abbott-Banner K., Poll C., Verkuyl J.M. (2013). Targeting TRP channels in airway disorders. Curr. Top. Med. Chem..

[47] Wallace H., Emir T.L.R. (2017). Airway Pathogenesis Is Linked to TRP Channels. Neurobiology of TRP Channels.

[48] Nicolson T.A., Bevan S., Richards C.D. (2002). Characterisation of the calcium responses to histamine in capsaicin-sensitive and capsaicin-insensitive sensory neurones. Neuroscience.

[49] Leason S.R., Barham H.P., Oakley G., Rimmer J., DelGaudio J.M., Christensen J.M., Sacks R., Harvey R.J. (2017). Association of gastro-oesophageal reflux and chronic rhinosinusitis: Systematic review and meta-analysis. Rhinology.

[50] Alnouri G., Cha N., Sataloff R.T. (2022). Histamine Sensitivity: An Uncommon Recognized Cause of Living Laryngopharyngeal Reflux Symptoms and Signs-A Case Report. Ear Nose Throat J..

[51] Bachert C., van Cauwenberge P., Olbrecht J., van Schoor J. (2006). Prevalence, classification and perception of allergic and nonallergic rhinitis in Belgium. Allergy.

[52] Pinzer T.C., Tietz E., Waldmann E., Schink M., Neurath M.F., Zopf Y. (2018). Circadian profiling reveals higher histamine plasma levels and lower diamine oxidase serum activities in 24% of patients with suspected histamine intolerance compared to food allergy and controls. Allergy.

[53] Hamada Y., Shinohara Y., Yano M., Yamamoto M., Yoshio M., Satake K., Toda A., Hirai M., Usami M. (2013). Effect of the menstrual cycle on serum diamine oxidase levels in healthy women. Clin. Biochem..

[54] Honzawa Y., Nakase H., Matsuura M., Chiba T. (2011). Clinical significance of serum diamine oxidase activity in inflammatory bowel disease: Importance of evaluation of small intestinal permeability. Inflamm. Bowel Dis..

[55] Kofler H., Aberer W., Deibi M., Hawranek T., Klein G.M., Reider N., Fellner N. (2009). Diamine oxidase (DAO) serum activity: Not a useful marker for diagnosis of histamine intolerance. Allergologie.

